# The Impact of COVID-19 from the Perspectives of Dutch District Nurses: A Mixed-Methods Study

**DOI:** 10.3390/ijerph182413266

**Published:** 2021-12-16

**Authors:** Jessica D. Veldhuizen, Sandra Zwakhalen, Bianca M. Buurman, Nienke Bleijenberg

**Affiliations:** 1 Research Centre for Healthy and Sustainable Living, Faculty of Health Care, University of Applied Sciences Utrecht, 3584 CS Utrecht, The Netherlands; 2 Department of Health Services Research, CAPRI Care and Public Health Research Institute, Maastricht University, Duboisdomein 30, 6229 GT Maastricht, The Netherlands; s.zwakhalen@maastrichtuniversity.nl; 3 Living Lab in Ageing and Long-Term Care, 6200 MD Maastricht, The Netherlands; 4 Department of Internal Medicine, Section of Geriatric Medicine, Amsterdam University Medical Center, University of Amsterdam, 1000 GG Amsterdam, The Netherlands; b.m.vanes@amsterdamumc.nl; 5 ACHIEVE, Centre of Applied Research, Faculty of Health, Amsterdam University of Applied Sciences, 1105 BD Amsterdam, The Netherlands; 6 Department of General Practice, Division Julius Center for Health Sciences and Primary Care, University Medical Center Utrecht, 3508 GA Utrecht, The Netherlands

**Keywords:** COVID-19, community health nursing, district nursing, home care, geriatric care, nursing research

## Abstract

Little is known about how COVID-19 affects older patients living at home or how it affects district nursing teams providing care to these patients. This study aims to (1) explore, from the perspectives of Dutch district nurses, COVID-19′s impact on patients receiving district nursing care, district nursing teams, and their organisations during the first outbreak in March 2020 as well as one year later; and (2) identify the needs of district nurses regarding future outbreaks. A mixed-methods, two-phase, sequential exploratory design was followed. In total, 36 district nurses were interviewed during the first outbreak (March 2020), of which 18 participated in the follow-up questionnaire in April 2021. Thirteen themes emerged, which showed that the COVID pandemic has substantially impacted patient care and district nursing teams. During the first outbreak, nurses played a crucial role in organising care differently and worked under high pressure, leading to exhaustion, tiredness, and psychosocial problems, including fear of infection. A year later, nurses were better prepared to provide COVID care, but problems regarding work pressure and mental complaints remained. The identified needs focus on a sustainable implementation of leadership roles for district nurses. At the organisational and national levels, more support and appreciation are needed in terms of trust and appropriate policies.

## 1. Introduction

COVID-19 has had a significant impact on healthcare systems worldwide [1]. In the short span of a few months, the number of deaths rose rapidly, and on 11 March 2020, the World Health Organization (WHO) declared COVID-19 to be a pandemic [2]. Globally, much attention has been given to the infection rates of COVID-19 patients in intensive care units (ICUs), hospitals in general, and long-term care facilities [3,4]. However, relatively little attention has been paid to how COVID-19 affects older patients living at home and to the homecare professionals who care for these patients in their own home [3].

Professional care assistance at home is provided through district nursing care and other healthcare professionals, such as general practitioners and (paramedic) professionals in primary care [5,6]. The organisation, delivery, and funding of district nursing care varies around the world [7,8,9]. In general, district nursing teams provide rehabilitative, preventive, or supportive care that covers all technical, psychosocial, and personal care to enhance peoples’ health and quality of life [8,10]. They visit many patients per day and provide nursing services and personal care services, such as assisting them with their medication intake, activities of daily living (ADLs), providing wound care, and supporting patients in the terminal phase of their lives [8,10].

During the COVID pandemic, district nursing teams worked on the front lines, visiting (frail) older people at home. This required considerable flexibility, creativity, and pragmatism in their work [3,4]. The teams played an essential role in detecting and preventing COVID-19 among high-risk patients and supporting those with confirmed or suspected COVID-19 who remained at home [11,12].

Some qualitative studies have described the impact of COVID-19 on home healthcare workers [11] and home health aides in the US [13]. Sterling et al. conducted 33 in-depth interviews with homecare workers in New York during the pandemic and found that home healthcare workers felt invisible despite working on the front lines [11]. They were scared due to the high risk of virus transmission, were forced to make difficult trade-offs, and received varying amounts of information, supplies, and training [11]. Another study by Osakwe revealed that home health aides experienced limited access to information, dilemmas related to enhanced COVID-19 precautions, and felt alone [13].

Despite the existence of studies such as those mentioned above, insights into the impact of COVID-19—specifically on those working in district nursing care—remain limited, especially for the situation in Europe and the Netherlands. It is unclear how COVID-19 has impacted district nursing teams and their organisations. Additionally, while some studies have described the impact of COVID-19 on community-dwelling older adults in general [14,15] or its effect on people with disabilities [16], the impact of COVID-19 on patients receiving district nursing care remains scarce. Therefore, this study focuses on how COVID-19 has affected the patients who are receiving and the nursing teams who are delivering district nursing care from the perspectives of district nurses.

Since the pandemic has changed across different waves, it is vital to understand how COVID-19′s impact among district nursing teams and patients has evolved over time. Little is known about how district nurses perceived the impact during and one year after the first outbreak and whether issues of concern have changed. Therefore, the aim of this study was to (1) explore, from the perspectives of Dutch district nurses, the impact of COVID-19 on patients who are receiving district nursing care, district nursing teams, and their organisations during the first outbreak in March 2020 as well as one year later and (2) to identify the needs of district nurses regarding future outbreaks.

## 2. Materials and Methods

### 2.1. Design

A mixed-methods study was performed in the Netherlands using two time frames, the first of which took place between March and May 2020 (first outbreak), and one a year later (April 2021). This mixed-methods study followed a two-phase, sequential exploratory design, in which the results of the first qualitative method (semistructured qualitative interviews) informed the second quantitative method (questionnaire) (QUAL → quan) [17].

### 2.2. Setting and Participant Selection

In the Netherlands, district nursing care is provided by teams of district nurses, vocational nurses, nursing assistants, and nursing aides (i.e., district nursing team). In 2018, 589,000 people received district nursing care (3.4% of the total population in the Netherlands), with a total cost of EUR 3.6 billion [18]. District nursing care was provided by 12,400 district nurses (European Quality Framework (EQF) level 5/6), 16,108 vocational nurses (EQF level 4), 41,799 nurse assistants (EQF level 3), and 4759 nurse aides (EQF levels 1 and 2) [19]. Other relevant positions within district nursing care in the Netherlands include specialised nurses, who have expertise in a specific topic (e.g., wound care) (EQF level 5/6), case managers for people with dementia (EQF level 5/6), and advanced nurse practitioners (EQF level 7). In general, the district nurse is in charge of care processes and assesses patient care needs and coordinates the patient’s care [10,20]. Because district nurses have been in charge of patient-related decision-making processes during the COVID-19 pandemic, district nurses were the target population of the current study.

District nurses establish organisational and professional ties with patients, informal caregivers, other healthcare professionals, local policy makers, and health insurers [21,22]. To equip and prepare district nurses to establish these connections, the Dutch Nurses’ Association developed a nationwide leadership programme for district nurses in the Netherlands [22]. This Dutch ambassador programme for district nurses is a 9-month leadership programme that started in 2013. Because district nurses who enrolled (and are presently enrolled) in this ambassador programme have more established organisational and professional links, only these nurses were invited to participate in the study. Up until March 2020, seven groups, comprising a total of 105 nurses, had finished this programme. The 105 nurses were contacted for the interviews via email. A convenience sample was used to select participants from this group. After receiving permission to participate, the interviewer contacted each nurse to provide more information about the study and to schedule an interview by phone.

For the follow-up questionnaire, the sample consisted of nurses who had participated in the previous interviews. No other nurses were invited to take part because the questionnaire also included a member check in which the participants reviewed the results of the first interviews. During this member check, the nurses checked if they agreed with the summary of the qualitative part of the study. Because the member check could only be conducted with previously participating nurses, only those who had participated in the interviews were contacted for the follow-up questionnaire. An additional reason for only contacting these nurses was to reduce the burden on all of the district nurses in the Netherlands. Finally, this mixed-methods study followed a QUAL → quan approach, and the emphasis of the current study lies on the qualitative part.

### 2.3. Data Collection

#### 2.3.1. Individual Interviews

The first detected COVID-19 patient in the Netherlands was reported on 27 February 2020, thus marking the start of the COVID-19 outbreak in the Netherlands. This outbreak continued until the end of May 2020, with a peak of 620 patients being admitted to the hospital per day on 27 March [23]. During this time, 16 interviewers with a background in nursing research and district nursing care held interviews in April and May 2020. Furthermore, NB, SMGZ, and BMB developed a semi-structured guide based on insights and experiences from district nursing practice (Appendix A). District nurses shared their experiences with the Dutch Nurses’ Association (V&VN) and the National Scientific Collaboration for District Nursing Care (in Dutch: Wetenschappelijke Tafel Wijkverpleging) during the COVID pandemic. This input was used to create the interview guide, which consisted of a protocol for the interviewer on how to conduct the interview, followed by questions regarding participant characteristics, the impact of COVID-19 on the nurse and nursing team, the impact on the patient, the effect on the organisation, current needs in district nursing care, and anticipated future challenges. All of the interviews were held by phone to reduce any burden on the nurse and to prevent the spread of COVID-19. The intended duration was 30 min. The interviews were recorded after receiving verbal permission from the nurse.

#### 2.3.2. Follow-Up Questionnaire

In April 2021, a year after the first outbreak, a link to an online questionnaire was sent by email to the nurses who participated in the interviews a year before. Due to high care demands during the intensive year for district nurses, a questionnaire was chosen instead of a follow-up interview. An online questionnaire was developed based on the results of the thematic analysis of the interviews. The results were presented in multiple themes. Per theme, three questions were asked: (1) “Do you recognise this description of the impact during the first COVID-19 outbreak in 2020?” (yes; no). If participants marked “no”, then they were asked to explain why they did not recognise the description. This question was asked as a member check of the themes that were analysed. To identify the impact of COVID-19 one year later, two additional questions were asked: (2) “How is the current situation in 2021′?”(improved; unchanged, deteriorated; improved and deteriorated) and (3) “Can you describe or explain the current situation?” (Appendix B). The first two questions were closed questions, whereas the third one was open. The questionnaire was distributed online using Qualtrics, an online survey platform [24]. The nurses were able to fill out the questionnaire between 1 and 30 April 2021. Two reminders were sent during this period to increase the response rate.

### 2.4. Data Analysis

#### 2.4.1. Interviews

Directly after the interview was held, the interviewer summarised the interview using the themes that were outlined in the interview guide. To check the validity, a nursing student (EQF level 6) compared the summaries to the recordings. No changes were made to the summaries. The summaries were then examined using a thematic analysis approach (Braun and Clarke, 2006), for which a nursing student (EQF level 6) and a researcher (JDV) independently coded and grouped them into categories and overarching themes. Differences were resolved by discussion until agreement was reached.

#### 2.4.2. Follow-Up Questionnaire

The first closed question regarding the member check was scrutinised using descriptive statistics (absolute numbers and percentages, means and standard deviations). Excel (version 2108, Microsoft Corporation, Washington, DC, USA.) was employed to calculate all of the descriptive statistics. In the open field of this question, the nurses had the chance to add or change information to describe the impact of the pandemic in. The open question where the nurses explained their perspectives was summarised, and minor changes were made to the results of the interviews (e.g., the results were more nuanced by adding words such as “often” or “sometimes”). The second closed question regarding the current situation was examined using descriptive statistics. The third open question was analysed using thematic analysis in the same way as stated above.

### 2.5. Ethical Considerations

Participation was completely voluntary. Because the nurses were not subjected to any actions, no ethical approval was needed under the Dutch law on medical research (WMO). However, all of the participants agreed to take part and provided consent, and permission to record the interview was obtained and recorded at the start of the interview. Additionally, the nurses gave their consent to participate in the follow-up questionnaire by ticking a corresponding box at the beginning of the questionnaire. The data were stored and examined based on the Dutch Personal Data Protection Act (AVG). Any personal details were removed from the questionnaire data to assure the anonymity of the data.

## 3. Results

Between April and May 2020, 105 district nurses were contacted to participate, of which 36 responded and were interviewed (34.3%). These nurses worked in 11 of the country’s 12 geographic areas (provinces); the mean years of work experience in district nursing care was 9.5 years (Table 1). In total, 34 nurses were contacted in April 2021 for the follow-up questionnaire. At the end of April 2021, 18 out of 34 district nurses had finished the questionnaire (53%). The mean duration of the interviews was 32 min.

Following the interviews, 13 themes were identified that described the impact of COVID-19 during the first outbreak in 2020 from the perspectives of the district nurses. The themes described (1) the downscaling and upscaling of district nursing care; (2), the changed daily care routine; (3) the impact on informal caregivers; (4) working with personal protective equipment (PPE); (5) increased work pressure; (6) fear of infection; (7) psychosocial effects and mental support; (8) leadership and the nurse’s role within the organization; (9) support from the organization; (10) uncertainty and worries about the future; (11) role and collaboration within district nursing care; (12) necessary changes for the future at the organizational and national levels; and (13) preparing for the future. These 13 themes were divided into three main themes: (1) impact on daily care for patients; (2) impact on district nursing teams; and (3) worries about and needs for the future.

**Figure 1:**
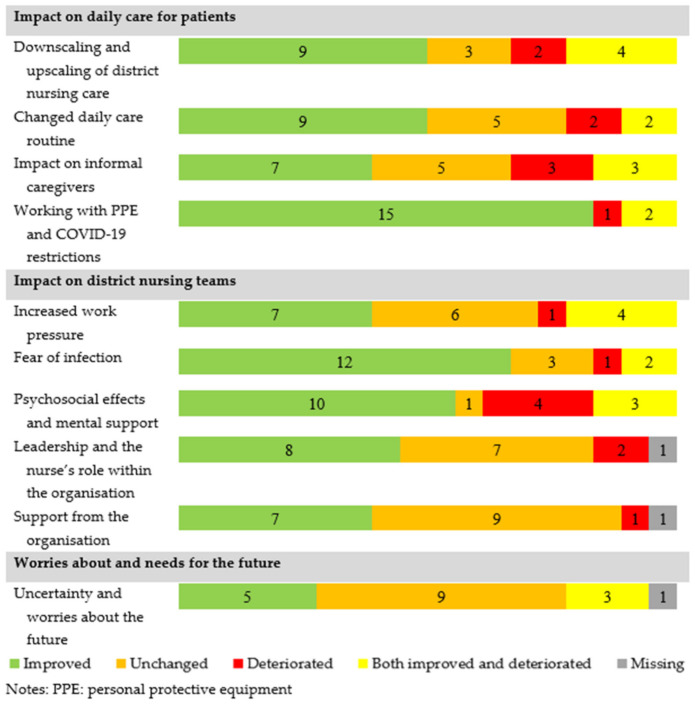
An overview of the changes in the impact of COVID-19 on district nursing care in 2021 a year after the first COVID-19 outbreak (2020) (N = 18).

A year after this first outbreak, the nurses were asked to reflect on the identified impact and whether the situation had improved, remained unchanged, deteriorated, or had both improved and deteriorated (Figure 1).

### 3.1. Impact on Daily Care for Patients

#### 3.1.1. Downscaling and Upscaling of District Nursing Care 

In 2020, nursing care for community-dwelling patients was often downscaled to a minimum (i.e., less care was provided to the patients) for three main reasons: (1) patients rejected care for fear of COVID-19 infection; (2) patients did not need care because of delayed operations; or (3) care could not be delivered due to the insufficient availability of nurses. Sometimes, more district nursing care was needed because nursing homes, outpatient clinics, and care organisations for social and day care were closed. Due to the downscaling of district nursing care, some patients learned to use healthcare aid devices and technology. 

A year later, most of the nurses found that the down- and upscaling of district nursing care improved (*n* = 9): Care at home had largely returned to its normal level, regular care by hospitals was continued (e.g., planned knee operations), and day care and nursing homes were opened again for frail older adults and people with dementia. Downscaling care at home was limited as much as possible, but it was sometimes still necessary given insufficient staffing levels and great care demands due to delayed or changed care. Care became more focused on the patient’s self-reliance and self-management compared to one year prior. According to the nurses, the (extra) use of healthcare technology should remain.

#### 3.1.2. Changed Daily Care Routine

During the first outbreak, care at home changed. District nurses experienced often that patients needed more psychosocial care or required (after) care due to COVID-19 infection. Potentially, there were fewer new patients in the picture because no physical home visits were allowed, and (video) calling was not always possible.

During the second outbreak, most nurses found that the changed daily care routine had improved (*n* = 9), while others found that it had remained unchanged (*n* = 5) or had deteriorated *(n* = 2). Indirect consequences such as loneliness and psychosocial problems among patients remained, but there was better support, and more attention was given to the patient’s needs. Patient visits remained limited. Overall, the alternation of digital and physical contact was (better) applied, and digital care consultations improved.

#### 3.1.3. The Impact on Informal Caregivers

In 2020, some informal caregivers provided less care due to fear of infection. Under other conditions, informal caregivers provided more care because they had more time available (e.g., they had lost their jobs or worked from home) or because informal care was needed due to the downscaling of care or the halting of day care activities for patients with dementia. This sometimes led to informal caregivers becoming stressed. The nurses experienced more contact and teamwork with informal caregivers.

A year later, the impact on informal caregivers was divided between improved (*n* = 7) and unchanged (*n* = 5) or deteriorated (*n* = 3). Sometimes, informal caregivers could and wanted to provide more care, which was partly due to the availability of PPE. At other times, the informal caregivers withdrew more for fear of infection or because they had less time available in their personal lives. Some caregivers were overworked and tired, with no space or time to recharge due to a lack of relaxation. There was still good contact and better cooperation with informal caregivers.

#### 3.1.4. Working with PPE and COVID-19 Restrictions

At the start of the COVID-19 pandemic, there were many questions about the safety of care at home. There was a lack of knowledge regarding PPE, and patients and nurses feared whether there was sufficient PPE available for safe care. Clear explanations and guidelines diminished this anxiety. However, the guidelines that were provided by the government or organisations were unavailable, unclear, or differed across organisations. Nurses and patients were frustrated by this lack of clarity. Some organisations had an acute shortage of PPE; other organisations had no deficits. The shortage of PPE felt there was less appreciation for district nursing care compared to other settings such as hospitals. With PPE, care was different and much more intense: Face masks (instinctively) provided distance and made it difficult for people to understand one another.

During the second outbreak, most nurses found that working with PPE had improved (*n* = 15): sufficient materials, documentation, and protocols were available. Nurses and patients were used to working with PPE. Sometimes, anxiety among patients and caregivers persisted despite the use of PPE. Vaccination, more knowledge, and being able to work preventively with PPE provided an increased feeling of safety. Face masks still created distance, and communication remained an obstacle. After one year, patients and informal caregivers had become tired of the COVID-19 situation. They did not see the seriousness of the situation, causing more laxity in testing, incorrect or no use of PPE, and less adherence to quarantine and other restrictions.

### 3.2. Impact on District Nursing Teams

#### 3.2.1. Increased Work Pressure

Depending on the number of COVID-19 infections among patients in 2020, the nurses had to work overtime, leaves of absence were withdrawn, and the nurses sometimes needed to be available and in action mode continuously. This time was experienced as a busy and chaotic period to work during because nursing student internships were halted, nurses became infected with COVID-19, or they needed to work on COVID teams. However, the “crisis mode” provided more challenge and creativity in their work, which was experienced as being positive. In areas with fewer COVID-19 infections, the downscaling of care at home sometimes led to more peace of mind at work.

A year later, the increased work pressure experienced in 2020 was equally divided between improved (*n* = 7) and unchanged (*n* = 6) or deteriorated (*n* = 1). On the one hand, less work pressure was experienced a year later due to fewer new patients, fewer infections among patients and district nursing professionals, the nurses having more free time available in their private lives, and people having gotten used to the circumstances. There was a feeling that the workload was better distributed within the organisation and a sense of balance was slowly returning. During quiet periods, there was room for leave among colleagues. On the other hand, more work pressure was experienced due to work overload and high absenteeism among district nursing personnel. This required considerable flexibility, which was experienced as tiring. The nurses ran on reserves, with insufficient space or time to recharge. They became emotional more quickly and wanted the situation to return to normal. The “action mode” was often still present.

#### 3.2.2. Fear of Infection

During the first outbreak, there was a fear of COVID-19 infection among nurses: They were afraid to infect or have infected patients and hence called in sick frequently. There was a sense of guilt and failure when dropping out because of an infection.

A year later, most nurses found that their fear of infection had improved (*n* = 12). Anxiety among healthcare providers had decreased because they were attuned, had improved knowledge, and had more experience working during the COVID-19 pandemic. Additionally, PPE was more (preventively) applied, and more patients, caregivers, and colleagues were vaccinated. Guilt for dropping out because of a (potential) infection was often still present.

#### 3.2.3. Psychosocial Effects and Mental Support

The nurses experienced stress, fear, and insecurity about the future during the first outbreak. Mental support was provided to patients and colleagues, and anxiety was managed, which took extra time and energy. Fellowship and solidarity between colleagues were increased, but this sometimes declined as physical meetings were not allowed.

When the second outbreak occurred, psychosocial effects and mental support tended to improve (*n* = 10), sometimes with deterioration (*n* = 4), or had improved and deteriorated (*n* = 3). Sometimes, stress and uncertainty were still present, but there was often less stress and work pressure because there were fewer COVID-19 cases. The feeling of powerlessness among the nurses was reduced due to more treatment options being available for patients infected with COVID-19. Often, solidarity, cooperation, and good initiatives prevailed among teams so that members supported each other and paid more attention to one another. Insufficient attention was given to indirect problems among district nursing staff due to the COVID-19 outbreak. There were more incidences of dropping out among district nursing staff due to mental complaints because of the long duration of the crisis.

#### 3.2.4. Leadership and the Nurse’s Role within the Organisation

The first COVID-19 outbreak demanded great leadership from nurses at different levels. Providing leadership to district nursing teams remotely was difficult because of missed signals. Nurses had to make choices regarding downscaling care, but it was sometimes difficult to make trade-offs (e.g., downscaling care or continuing care with risks for patients and the district nursing team). According to some nurses, there was a return to old structures, with more hierarchy and less flexibility, with managers making decisions without nurses being involved. Additionally, management often stopped including nurses in policy matters (e.g., projects within or between organisations). It was sometimes a struggle to act autonomously as a nurse. For other nurses, decision-making processes within the organisation were faster, and more choices were made in a bottom-up fashion by nurses instead of through a top-down approach by management.

A year later, experiences regarding leadership and the nurse’s role varied; it was often unchanged (*n* = 7) or deteriorated (*n* = 2), but for some nurses, it had improved (*n* = 8). District nurses still had to make difficult choices. In some cases, district nurses were not involved in the decisions that were made by their managers, and they experienced little room to voice their opinions. Additionally, organisations were structured with more hierarchy. In other cases, there was similar or more attention given to the autonomy and leadership of district nurses, and some nurses had leeway to participate in other projects. There was more time for the nurses to do what they are good at, and district nursing teams were completely self-managed once more.

#### 3.2.5. Support from the Organisation

During the first outbreak, nursing organisations provided various prerequisites such as necessary materials (e.g., PPE, thermometers, tablets), study time to enhance knowledge, testing options for district nursing staff, emotional support and psychological assistance, and childcare for district nursing staff. There was gratitude, appreciation, and recognition for district nursing teams; for example, they received small gifts such as flowers and compliments from management. The ways in which organisations communicated COVID-related information was conducted differently (e.g., one organisation set up a helpline for questions staffed by district nurses; elsewhere, managers were constantly available and focused on eliminating knowledge deficits). Communication within organisations was experienced differently, from “poor” to “excellent”.

During the second outbreak, most nurses found that support from their organisation had improved *(n* = 7) or remained unchanged (*n* = 9). Support and appreciation often improved or remained the same. Support was experienced as pleasant and fit well with what was needed. Manager communication and accessibility had improved. A few nurses mentioned that management showed insufficient attention.

### 3.3. Worries about and Needs for the Future

#### 3.3.1. Uncertainty and Worries about the Future

In 2020, the nurses experienced uncertainty and were concerned about the future related to the use of care (e.g., postponed hospital admissions, closed day care), the costs of care (costs for purchasing needed materials and reimbursement for non-provided care), and how care could stay patient-centred with attention given to frail older adults.

In 2021, most nurses still experienced uncertainty and worries about the future (*n* = 9). They explained that they were more prepared than they had been one year prior and knew how to identify and treat someone with COVID-19. However, uncertainty remains about the long-term consequences of COVID-19 for patients, district nursing teams, and organisations.

#### 3.3.2. Future Role and Collaboration within District Nursing Care

During the first outbreak, the nurses felt that district nursing leadership must be maintained in the future with respect to their professional autonomy. A balance should be sought in restarting care by looking critically at building up and scaling down needed care. In this regard, more attention should be paid to the patient’s self-reliance and self-management, eliminating unnecessary care, the use of informal care, and the use of healthcare aid devices and technology. The COVID-19 pandemic showed that district nursing care could manage complex forms of care such as transmural care, acute care at home, and complicated wound care. More could be implemented to improve collaboration across the boundaries of organisations.

**Figure 2:**
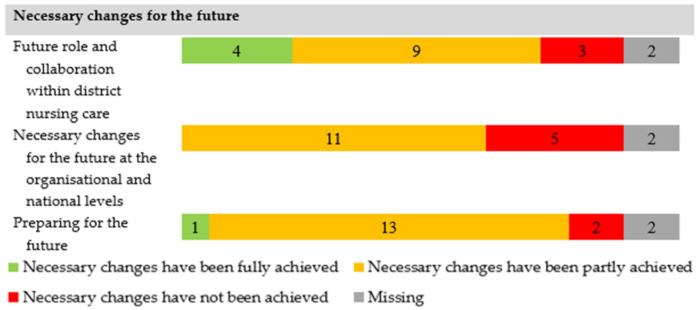
Necessary changes for the future identified in 2021, a year after the first outbreak (2020) (N = 18).

During the second outbreak, most nurses found that necessary changes for the future had been fully (*n* = 4) or partly achieved (*n* = 9) (Figure 2). Three nurses found that the required changes had not been achieved. The nurses experienced insufficient room to focus on other tasks, such as prevention or improving district nursing care. Cross-organisational collaboration in primary care often improved but was found to be declining once again in some cases. Cooperation with hospitals and intramural institutions was enhanced. The nurses hoped that collaboration would continue to exist.

#### 3.3.3. Necessary Changes for the Future at the Organisational and National Levels

In 2020, the nurses mentioned that fundamental changes were needed at the organisational and national levels. The nurses stated that more attention should be given to district nursing care, especially regarding the safety of care and loneliness among patients during the pandemic. They also stated that the government and insurers should also provide more support, attention, and appreciation for district nursing care. The nurses mentioned that a specific policy for district nursing during the pandemic was desirable. A national knowledge platform to share knowledge would be helpful, and guidelines should be better translated to district nursing. Nurses perceived that more research should be conducted and shared regarding COVID-19 (e.g., recognising signals, evaluation of past periods) in district nursing care.

One year later, the nurses found that necessary changes at the national level had been either partially (*n* = 11) or not achieved (*n* = 5). The importance of nurses has become more visible. However, there is still a need to bring the impact of COVID in district nursing to the attention of the general public. District nurses must show more leadership and make themselves heard. Nurses wish for more appreciation for district nursing in the form of higher salaries and more confidence in district nursing as a profession. According to the nurses, the government and insurers must provide more time and financial resources.

#### 3.3.4. Preparing for the Future

In 2020, the nurses mentioned that a (national) plan for new COVID-19 outbreaks is desirable to provide safe and responsible care. Care tasks surrounding the patient (e.g., prevention at the community level) should be resumed instead of (only) focusing on the primary process. Additionally, care pathways and rehabilitation processes should be established for ex-COVID patients.

One year later, most nurses found that the necessary changes had been partially achieved (*n* = 13). Solid plans and guidelines are available for new outbreaks; however, working in district nursing care requires customising care to specific patients. The nurses stated that they had learned a lot in a short period and did many things well. It is unclear how nurses should deal with the overall damage from the pandemic. The care path for ex-COVID patients still requires improvements.

## 4. Discussion

To the best of our knowledge, this is the first mixed-methods study that describes the impact of the COVID-19 pandemic on district nursing care from the perspectives of Dutch district nurses. Our study shows that the pandemic has had a deep impact on patients, informal caregivers, and district nursing teams. Care for patients has changed, and together with the patient and informal caregivers, nurses have often experienced more work pressure and more psychosocial problems, including a greater fear of infection. The role of the district nurse as a leader has changed substantially. The study also identified multiple needs for the future, where more focus should be placed on the role of nurses, necessary changes at the organisational and national levels, and how district nursing teams can be better prepared for the future.

While nurses in district nursing care generally experienced high work pressure prior to the COVID-19 pandemic [25], even greater work pressure was experienced during the first outbreak due to providing COVID care in the community, combined with more care at home because nursing homes, outpatient clinics, and care organisations for social and day care were closed down. On the other hand, care for patients at home was often downscaled due to fear of infection, delayed operations, or insufficient availability of nurses. This has been seen in other studies as well [26,27]. This shift in care delivery at home had a high impact on informal caregivers, sometimes leading to informal caregivers becoming stressed during the first outbreak as well as a year later. Chan et al. stated that informal caregivers are the “forgotten healthcare workers during the COVID-19 pandemic” and emphasised that urgent research is needed in district nursing care to support health needs during extreme events such as the COVID-19 pandemic [28]. The lack of knowledge, guidelines, and materials such as PPE during the first months made district nurses feel less appreciated as a nursing profession. This was seen not only in the Netherlands, but in other countries as well [11,26,29]. The change in workload for nurses had a deep impact on their wellbeing and mental health; they experienced stress, fear, and insecurity. Those working in district nursing care and other healthcare workers were at high risk for mental problems and burn-out, especially during the COVID-19 pandemic [30,31,32]. This requires additional efforts at the organisational and national levels to support district nursing teams [11,30,33,34].

One year after the first outbreak, the nurses perceived that significant improvements had been made regarding PPE, as more materials were available. There was less fear of infection due to sufficient PPE and the availability of vaccinations. Support from organisations had also improved or remained unchanged, which was a good thing since most nurses were optimistic about support in 2020. On the other hand, the impact on caregivers and the effect on time and energy among nurses were often unchanged or had even deteriorated. These caregivers and nurses were overworked and tired, with no ability to recharge due to a lack of time to relax. However, some nurses experienced less work pressure because of fewer new patients, fewer infections among patients and district nursing staff, and more available free time in their personal lives. Psychosocial effects and mental support had sometimes improved; however, some nurses experienced deterioration. The nurses felt that insufficient attention was given to indirect problems among nurses. Due to the long duration of the crisis, there were more incidences of dropping out among nurses due to complaints about mental health.

Looking at needs during the first outbreak and one year later, most change is needed regarding the role of nurses and support and trust at the national level. District nursing is marked as a specialty nursing practice at the national [35] and international levels [36,37,38,39], with specific nursing interventions and competencies. At the national level, the ambassador’s project was developed to strengthen district nurses’ leadership skills [22]. Although the importance of district nurses has become more visible among the public, they wish for more confidence in district nursing as a profession. During the first outbreak, the nurses experienced a return to old structures with more hierarchy, less flexibility, and insufficient leeway to focus on other tasks, such as policy developments or quality improvements. The nurses desire more trust, support, attention, and appreciation by health insurers and the government. Additionally, the nurses felt that their role as a nursing leader, having a crucial responsibility both for team members (i.e., translating guidelines and supporting others) and patients (organising care), must be maintained concerning their professional autonomy. In terms of the care provided, the pandemic has shown that district nurses are able to manage complex care (such as transmural care, acute care at home, and complicated wound care), and the nurses wish to continue doing so. This indicates a district nurse can fulfil the specialist-generalist role [40]. During the first wave of the pandemic, the nurses experienced improved cross-organisational collaboration. However, one year later, this sometimes declined again. Additionally, the nurses felt that cooperation with hospitals and other care facilities could be improved. The lack of cooperation can be explained by the organisation of district nursing practice in the Netherlands, in which district nursing care is provided by 3070 different care organisations [18]. More uniformity among district nursing care organisations could help improve collaboration among the various care providers.

Initially, this study aimed to identify how the pandemic has affected organisations and organisational choices. This question was part of the interview guide. However, after analysing the results, it was decided to place the answers under other themes since they fit better under such themes (e.g., the upscaling and downscaling of care under the impact of daily care for patients, and support from the organisation under the impact on district nursing teams). This study focused on the perspectives of district nurses during the COVID-19 pandemic, in which they fully focused on organising and providing care for patients and their teams. This may explain why the effects at the organisational level were not as visible for the nurses. To identify the impact of COVID-19 at the organisational level, additional research conducted with managers of organisations would be helpful.

### 4.1. Strengths and Limitations

This study rapidly identified the experiences and impact of COVID-19 on district nursing care during the first outbreak. Nurses from multiple organisations across the Netherlands were included. Additionally, the mixed-methods design provided us with valuable insights into the experiences of district nurses during the pandemic over time. Furthermore, the results of the interviews were checked by those who responded to the questionnaire as a member check.

While the number of respondents for the interviews was sufficient and the response rate to the follow-up questionnaire was relatively high (53%), the total number of district nurses participating one year later with the questionnaire was low. One possible reason for the nurses not participating in the questionnaire could be that they did not remember the interviews. The low number of participants (N = 18) in the quantitative part of the study makes it problematic to generalize the findings nationwide. The results of the study should therefore be carefully interpreted. In addition, we selected a specific group of district nurses who took part in the Dutch district nurses ambassador’s leadership programme. This programme includes nurses who are motivated to participate in that programme, making it difficult to generalise the findings for all nurses. Finally, it is possible that the identified themes were prompted by the interview guide, which also focused on the impact on the patient, nursing teams, and at the organisational level. However, in this study, following the results of the interviews, it was decided not to create a special theme regarding the impact at the organisational level since not enough input regarding the impact of COVID on the organizational level was identified.

### 4.2. Implications for Practice, Policy and Research

The results of our study show that district nurses have played a crucial role during the pandemic, and not only in direct patient care; nurses have supported their team members and have played a significant role in their organisations, translating policy guidelines into practical ones. The nurses have become able to handle complex care and set up different workarounds and innovative collaboration among various organisations in their working area. The nurses highlighted that this role should be maintained after COVID-19. Moreover, organisations should constantly foster nurse leadership and invite district nurses to the table to discuss organisational matters more often. At the same time, nurses should be proactive and take the opportunity to assume their role. At the policy level, more attention should be given to the vital work of district nursing. There is a pressing need for the importance of district nursing care to be recognised, prioritised, and adequately resourced at the organisational and national levels [3,4,41]. Moreover, to be better prepared for future pandemics and the current demographical and societal challenges that disrupt healthcare service delivery, a solid evidence base for district nursing care is required [9].

## 5. Conclusions

This study shows that the COVID-19 pandemic has substantially impacted patient care and professionals in district nursing care. Nurses have played a crucial role in organising care differently and have worked under high pressure, leading to exhaustion, tiredness, and psychosocial problems, including a fear of infection. While nurses have become better prepared to provide COVID-19 care after one year, change is still needed, especially regarding the sustainable implementation of leadership roles for district nurses within and outside their respective organisations to enhance district nursing practice for patients and professionals. Additionally, more support and appreciation are needed in terms of trust and appropriate policies at the organisational and national levels.

## Figures and Tables

**Table 1:** Demographic characteristics of interview and follow-up questionnaire participants.

	Interviews (2020) N = 36	Follow-Up Questionnaire (2021) N = 18
Age; mean (sd)	43.0 (12)	42.5 (10.3)
Sex: female; n (%)	33 (91.7)	18 (100)
Function; n (%)	19 4 1 2	14 3 1 0
– District nurse – Case manager for people with dementia – Advanced nurse practitioner (in training) – Other (e.g., specialised wound care nurse, short-term care stay nurse)		
Years of experience in district nursing care; mean (SD)	9.5 (5.2)	14 (7.0)

## Data Availability

The data presented in this study are available upon request from the corresponding author. The data are not publicly available due to privacy restrictions (i.e., containing information that could compromise research participant privacy).

## Appendix A. Interview Guide

**General Questions**
● Are you currently working in district nursing care? If not, where are you working? If not related to district nursing care, finish the interview
● At what organisation are you working?
● For how long have you been working in district nursing care?
● What is your function title?
● How old are you?
**Specific Questions Regarding the Impact of COVID-19 on District Nursing Care**
● What impact have you experienced as a result of the COVID-19 crisis?
● What is the impact of COVID-19 on the client?
● What is the impact of COVID-19 on the organisation and organisational decisions?
● Are you well equipped in your work in district nursing care to properly perform your role during the COVID-19 crisis? Please explain.
● Where are the greatest needs in district nursing care at this moment (during the COVID-19 crisis)?
● What challenges do you foresee for yourself, the client, and the organisation in the near future?
● What do you need and from whom to respond to these challenges?

## Appendix B. Online Questionnaire

General questions

**Question**	**Answer Options**
What is your gender?	Male Female I’d rather not say
How old are you?	<open field>
What is your current function?	District nurse (bachelor’s degree required) Specialised nurse Nurse specialist Other <open field>
What is your education level?	Bachelor’s degree Master’s degree at a university for applied science Master’s degree in education at university
How many hours per week do you work in district nursing care?	<open field>
How long have you been working in district nursing care?	<open field>

Specific questions for the first 10 subthemes (the downscaling and upscaling of district nursing care; changed daily care routine; the impact on informal caregivers; working with personal protective equipment (PPE) and COVID-19 restrictions; increased work pressure; fear of infection; psychosocial effects and mental support; leadership and the nurse’s role within the organisation; support from the organisation; uncertainty and worries about the future).

**Question**	**Answer Options**
Do you recognise the above description of [the subtheme] in district nursing care during the first outbreak? If not, please explain	Yes No <open field>
How is the current situation of downscaling in district nursing care?	Situation is improved Situation is unchanged Situation is deteriorated Situation is both improved and deteriorated
Please explain	<open field>

Specific questions for the last three subthemes (future role and collaboration within district nursing care; necessary changes at the organisational and national levels; preparing for the future).

**Question**	**Answer Options**
Do you recognise the above description of [the subtheme] in district nursing care during the first outbreak? If not, please explain	Yes No, <open field>
Has the situation regarding your future role and collaboration in district nursing care already been realised or implemented?	Yes Yes, partly No
Please explain	<open field>

Final questions

**Question**	**Answer Options**
What has helped you the most during the COVID-19 crisis in district nursing care?	<open field>
What has bothered you the most during the COVID-19 crisis in district nursing care?	<open field>
This is the final question. If there is anything else you would like to say about this subject or research, you can do so here.	<open field>
